# Design, synthesis, *in silico*, and *in vitro* evaluation of benzylbenzimidazolone derivatives as potential drugs on α-glucosidase and glucokinase as pharmacological targets†

**DOI:** 10.1039/d3ra02916f

**Published:** 2023-07-12

**Authors:** Cress Lumadhar Santos-Ballardo, Julio Montes-Ávila, José Guadalupe Rendon-Maldonado, Rosalio Ramos-Payan, Sarita Montaño, Juan I. Sarmiento-Sánchez, Selene de Jesús Acosta-Cota, Adrián Ochoa-Terán, Pedro de Jesús Bastidas-Bastidas, Ulises Osuna-Martínez

**Affiliations:** a Facultad de Ciencias Químico Biológicas, Universidad Autónoma de Sinaloa México ulises.osuna@uas.edu.mx jmontes@uas.edu.mx; b Facultad de Ingeniería Civil, Universidad Autónoma de Sinaloa México; c Departamento de Ciencias de la Salud, Universidad Autónoma de Occidente México; d Centro de Graduados e Investigación en Química, Instituto Tecnológico de Tijuana México; e Laboratorio de Análisis de Residuos de Plaguicidas, Centro de Investigación en Alimentación y Desarrollo A.C. México

## Abstract

Benzimidazolones have shown biological activities, including antihyperglycemic and hypoglycemic, by inhibiting or activating of α-glu and GK. The aim of this study is the rational design of compounds using *in silico* assays to delimitate the selection of structures to synthesize and the *in vitro* evaluation of benzimidazolone derivatives in blood glucose control. A docking of 23 benzimidazolone derivatives was performed; selecting the compounds with better *in silico* profiles to synthesize by microwave-irradiation/conventional heat and evaluate in enzymatic *in vitro* evaluation. Compounds 2k, 2m, 2r, and 2s presented the best *in silico* profiles, showing good affinity energy (−10.9 to −8.6 kcal mol^−1^) and binding with catalytic-amino acids. They were synthesized at 70 °C and 24 h using DMF as the solvent and potassium carbonate (yield: 22–38%). The results with α-glu showed moderate inhibition of 2k (14 ± 1.23–29 ± 0.45), 2m (12 ± 2.21–36 ± 0.30), 2r (7 ± 2.21–13 ± 1.34), and 2s (11 ± 0.74–35 ± 2.95) at evaluated concentrations (0.1 to 100 μg mL^−1^). The GK activation assay showed an enzymatic activity increase; compound 2k increased 1.31 and 2.83 more than normal activity, 2m (2.13-fold), 2s (2.86 and 3.74-fold) at 100 and 200 μg mL^−1^ respectively. The present study showed that the 2s derivative presents moderate potential as an α-glu inhibitor and a good activator potential of GK, suggesting that this compound is a good candidate for blood glucose control through antihyperglycemic and hypoglycemic mechanisms.

## Abstract 2

An *in silico* assay was designed, and generated compounds were studied to delimitate the synthesis by microwave irradiation–conventional heat combination. *In vitro* evaluation as antihyperglycemic and hypoglycemic agents were made, where 2s has good potential.

## Graphical abstract

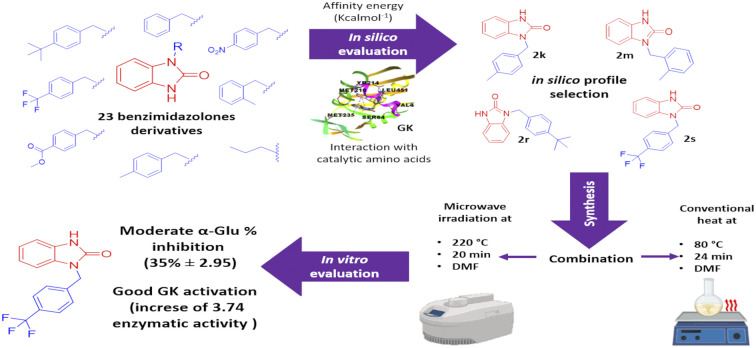

## 1 Introduction

Benzimidazolones are heterocyclic compounds that have shown many biological activities, including antiallergic, antiemetic, antipsychotic, antibacterial, antifungal, antiviral, antihyperglycemic, and hypoglycaemic.^1–5^ Because of this, some authors reported the synthesis of these compounds using methods like 1,3-dipolar cycloaddition, condensation, cycloisomerization, and direct functionalization. However, these routes could be expensive, and sensitive metallic catalysts or harsh reaction conditions are needed.^6^ The use of a combination of alternative energy sources like microwave irradiation and conventional heat could help to reduce reaction time and generate benzimidazolone derivatives with biological potential. The use of benzimidazolone derivatives has been reported for blood glucose control since they can either lower these levels (hypoglycemic) or slow the absorption of the molecule (antihyperglycemic) by the inhibition or activation of pharmacologic targets like α-glucosidase (α-Glu) and glucokinase (GK).^7,8^ The intestinal enzyme α-Glu, belongs to the glucoside hydrolase family, and performs the hydrolysis of the α 1,4-glycosidic bonds of the dietary carbohydrates; this action generates glucose absorption and their distribution to the bloodstream.^9^ α-Glu is located in the brush border of the enterocytes and is considered the final step of carbohydrates metabolism because it catalyzes the hydrolysis of α-glycosidic linkages of disaccharides and oligosaccharides generated glucose directly into the bloodstream. Based on its action mechanisms, this enzyme is closely associated with some metabolic diseases; through the stimulation of glucose absorption in patients with a high blood level of this molecule, promoting hyperglycemic states.^10–12^ Therefore, some α-Glu inhibitor were designed and evaluated *in silico* and *in vitro*; for example, *in silico* assays have shown that inhibitors interact with important amino acids residues like Phe178, Phe303, His280, His351, Arg315, Arg442 and Tyr158.^13^ Also, some studies have shown good inhibition against this intestinal enzyme by synthetic heterocyclic compounds like pyrrole and pyrrolidine derivatives, indole derivatives, and benzimidazolone derivatives with inhibitory concentration 50 (IC_50_) values of 0.77–4.1, 5.4–7.1 and 2.2–87 μM respectively.^14,15^ On the other hand, GK is a pharmacologic target that promotes insulin secretion in pancreatic β-cell to regulate blood glucose levels; this mechanism involves the closure of the potassium channels (KATP), which stimulates the released insulin into the bloodstream.^16,17^ Due to these actions and the relevance of generating new pharmacologic compounds in blood glucose control, the study and evaluation of GK as a novel pharmacologic target is important; some *in vitro* studies have been reported; Ren *et al.* in 2021 demonstrated the relevance of interaction with Thr65, Tyr214, and Val455 in the allosteric site in GK, suggesting this site in the enzyme important in the increase on the enzymatic activity.^18^ Recently, small molecules were evaluated as new allosteric site activators of this enzyme, for example, dorziaglatin is currently being investigated in phase III clinical trials, while PB-201 and AZD-1656 have reached phase II clinical trials.^19–21^ Also, quinazolin-4-one derivatives were designed and evaluated *in silico* and *in vitro* against GK, this study presented an effective concentration 50 (EC_50_) from 382–1870 nM.^22^ The using of *in silico* studies can help to delimitate the generation and evaluation of biologically active compounds; within the main *in silico* method used in this process is the molecular docking, which is a simulation of the interaction between a ligand and protein, showing values of affinity energy and the interaction with amino acid residues in the union site.^23^ To corroborate the molecular docking interactions, the *in vitro* evaluation is necessary; to determine the inhibitory (α-glu) or activator (GK) effect of biologically active compounds like benzimidazolones derivatives, specifically as blood glucose control drugs. The aim of this study is the rational design of benzimidazolones derivatives, using *in silico* evaluation to determine the interaction against these pharmacologic targets and delimitate the selection of structures to synthesize under microwave irradiation/conventional heat conditions, and evaluate with *in vitro* inhibition of α-glu and the activation of GK.

## 2 Experimental

### 2.1 Design of benzimidazolones derivatives for *in silico* assays

23 Benzimidazolones derivatives (2a–w) were designed, using electron withdrawing groups and functional groups as aliphatic, halogens, aromatic rings, nitrogen, oxygen, *etc.* (Table 1), to generate interactions like polar hydrogen, electrostatic and hydrophobic bonds with catalytic or relevance amino acid in the union site of the pharmacologic target.^11^

**Table 1:** List of radicals in benzimidazolone derivatives to evaluate an *in silico* molecular docking

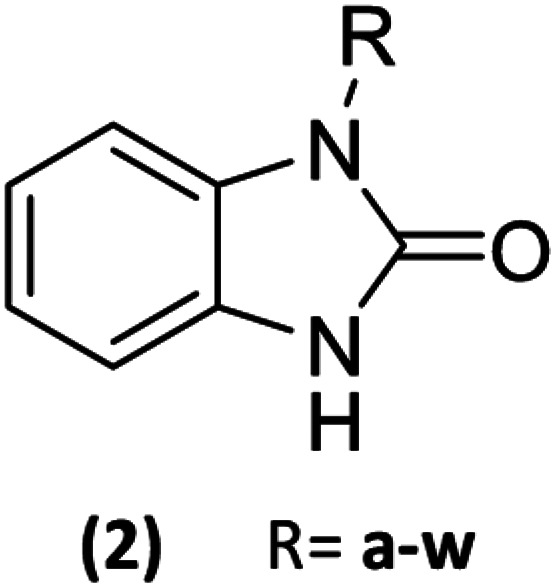
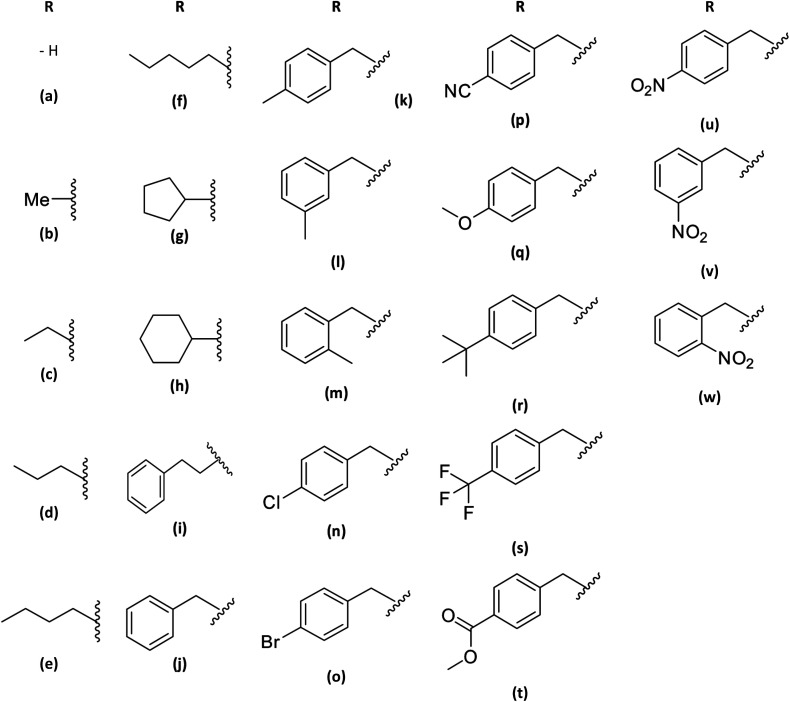

### 2.2 Molecular docking

The molecular docking studies were carried out using Autodock Vina and were guided to the pharmacologic targets active (α-glu) and/or allosteric site (GK). The benzimidazolones derivatives were designed using ChemDraw V16. The 3D optimization was done in Spartan V14 using MMFF94’s where Gasteiger charges were assigned to the ligands. The crystal structures for human lysosomal acid α-glucosidase (PDB: 5NN8) and human glucokinase (PDB: 3IMX) were downloaded from the RCSB protein data bank. All water, and ligands, were removed from the crystal receptors to perform the docking. Then, polar hydrogens and Kollman charges were assigned. The grid box was set for α-glucosidase with a grid of 74, 70, and 90 points (*x*, *y*, and *z*) with a grid spacing of 0.375 Å. case of GK allosteric site with a grid of 66, 62, and 40 points with a grid spacing of 0.375 Å. Docking calculations were performed using the Lamarckian genetic algorithm for ligands conformational searching with 200 runs with a population size of 150 individuals, a maximum of 25 million energies evaluations, a maximum of 270 000 generations, a gene mutation rate of 0.02 and a crossover rate of 0.8. Cluster analysis was performed on the docked results using a root mean square (RMS) tolerance of 0.5 Å. The docking conformation with the lowest binding energy was examined and visualized with Accelrys Discovery Studio v17.2.0.16349 [Accelrys Inc., San Diego, CA (2007)].^24,25^

### 2.3 General procedure for the benzimidazolone derivatives synthesis (2k, 2m, 2r and 2s)

**Fig. 1:**
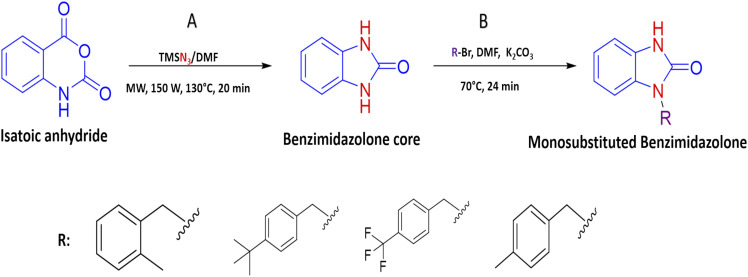
Scheme of synthesis of benzimidazolone derivative a combination of microwave irradiation and conventional heat: (a) in the first part of synthesis, it used isatoic anhydride as limiting compound, trimethyl silane azide and DMF in microwave conditions to generate the benzimidazolone core. (b) Later, this compound was use in alkylation with benzyl bromide in conventional heat conditions to generate benzimidazolones monosubstituted.

The synthesis of benzimidazolone core (2a) was performed using microwave irradiation using isatoic anhydride as the limiting compound. To a microwave reactor vessel (10 mL) were added an isatoic anhydride (1.0 mmol), trimethyl silane azide (1.4 mmol) and *N*,*N*-dimethylformamide (DMF) (2.0 mmol). The mixture was heated at 130 °C for 20 min and then was cooled to room temperature. In the resulting mixture, i-PrOH (2 mL) was added and shaken to precipitate the fine solid formed. After that, the liquid was removed and the solid was dried at room temperature to obtain the benzimidazolone core (2a) in high purity. Characterization data are similar to those reported in the literature. In order to generate the benzimidazolones alkylated, to a flat-bottomed flask was added 2a (1.5 mmol), potassium carbonate (1.0 mmol), a series of benzyl bromides (1.5 mmol) to introduce these groups in the heterocyclic nitrogen, and *N*,*N*-dimethylformamide (DMF) as solvent (2 mmol). The mixture was heated at 70 °C for 24 hours. After the reaction time, ethyl acetate (EtOAc) was added (15 mL); subsequently, two washes were made by adding cool water (15 mL), mixing, and reserving the organic phase. The resulting mixture was concentrated by rotavapor and purified by chromatographic column (hexane/EtOAc 7 : 3 v/v) to obtain the benzimidazolones derivatives in high purity (Fig. 1).

#### 2.3.1 1-(4-Methylbenzyl)-1,3-dihydro-2*H*-benzo[*d*]imidazol-2-one (2k)

White solid, yield 38% (100 mg, 0.4 mmol), M.p. 190–194 °C. IR (KBr disk, cm^−1^) 3360, 3150, 2928, 1702, 1483, 1151; ^1^H-NMR (400 MHz, CDCl_3_): *δ* 10.09 (s, 1H, NH), 7.14–7.17 (m, 2H, H-Ar), 7.02–7.05 (m, 3H, H-Ar), 6.88–6.97 (m, 2H, H-Ar), 6.78–6.80 (m, 1H, H-Ar), 4.98 (s, 2H, CH_2_-Ar), 2.23 (s, 3H, CH_3_-Ar). ^13^C-NMR (100 MHz, CDCl_3_): *δ* 156.0 (C=O), 137.5, 133.4, 130.4, 129.6, 128.2, 127.5, 121.8, 109.8, 108.7, 44.5 (CH_2_-Ar), 29.9, 21.2 (CH_3_-Ar); GC-MS (CI^+^) *m*/*z*: 238.2 u. [M + H]^+^.

#### 2.3.2 1-(2-Methylbenzyl)-1,3-dihydro-2*H*-benzo[*d*]imidazol-2-one (2m)

White solid, yield 23% (44.6 mg, 0.2 mmol), M.p. 158–160 °C. IR (KBr disk, cm^−1^) 3195, 3064, 2913, 1700, 1490, 1143; ^1^H-NMR (400 MHz, CDCl_3_): *δ* 10.13 (s, 1H, NH), 7.11–7.10 (m, 2H, H-Ar), 7.06–7.04 (m, 2H, H-Ar), 6.98–6.94 (m, 2H, H-Ar), 6.91–6.86 (m, 1H, H-Ar), 5.02 (s, 2H, CH_2_-Ar), 2.33 (s, 3H, CH_3_-Ar). ^13^C-NMR (100 MHz, CDCl_3_): *δ* 156.0 (C=O), 135.9, 133.8, 130.7, 130.5, 128.2, 127.7, 126.9, 121.9, 121.5, 109.8, 108.9, 43.0 (CH_2_-Ar), 29.9, 19.3 (CH_3_-Ar); GC-MS (CI^+^) *m*/*z*: 238.1 u. [M + H]^+^.

#### 2.3.3 1-(4-(*ter*-Butyl)benzyl)-1,3-dihydro-2*H*-benzo[*d*]imidazol-2-one (2r)

White solid, yield 26% (65.1 mg, 0.2 mmol), M.p. 185–188 °C. ^1^H-NMR (400 MHz, CDCl_3_): *δ* 10.31 (s, 1H, NH), 7.26–7.24 (m, 2H, H-Ar), 7.20–7.17 (m, 2H, H-Ar), 7.05–7.03 (m, 1H, H-Ar), 6.97–6.90 (m, 2H, H-Ar), 6.84–6.82 (m, 1H, H-Ar) 4.99 (s, 2H, CH_2_-Ar), 1.20 (s, 9H, *ter*Bu-Ar). ^13^C-NMR (100 MHz, CDCl_3_): *δ* 156.1 (C=O), 150.8, 133.4, 130.4, 128.3, 127.2, 125.8, 121.8, 121.4, 109.9, 108.7, 44.3 (CH_2_-Ar), 34.6, 31.4 (C(CH_3_)_3_); GC-MS (CI^+^) *m*/*z*: 280.2 u. [M + H]^+^.

#### 2.3.4 1-(4-(Trifluoromethyl)benzyl)-1,3-dihydro-2*H*-benzo[*d*]imidazol-2-one (2s)

White solid, yield 22% (37.8 mg, 0.1 mmol), M.p. 185–188 °C. IR (KBr disk, cm^−1^) 3191, 2939, 1706, 1396, 1124; ^1^H-NMR (400 MHz, CDCl_3_): *δ* 8.32 (s, 1H, NH), 5.61–5.59 (m, 2H, H-Ar), 5.47–5.45 (m, 2H, H-Ar), 5.16–5.02 (m, 3H, H-Ar), 3.18 (s, 2H, CH_2_-Ar). ^13^C-NMR (100 MHz, CDCl_3_): *δ* 155.9 (C=O), 140.3, 130.1, 130.0, 128.2, 127.7, 126.0, 125.9, 122.2, 121.7, 110.1, 108.4, 44.2 (CH_2_-Ar); GC-MS (CI^+^) *m*/*z*: 292.1 u. [M + H]^+^.

### 2.4 α-glucosidase inhibitory activity

The α-glucosidase (EC 3.2.1.20) activity of the synthetic benzimidazolones derivatives was assessed as described by Aispuro-Perez *et al.* and Santos-Ballardo *et al.,* in 2020, with slight modification.^25,26^ In a 96-well flat-bottom plate, 100 μL of the enzyme solution (1 U mL^−1^ in 0.1 M of phosphate buffer at pH 7.0) and 50 μL of benzimidazolones derivatives 2k, 2m, 2r, and 2s (concentrations of 0.1, 1, 10, 50, 100 μg mL^−1^ in DMSO 10% in methanol) were added. After 10 min of incubation at 25 °C, 50 μL of 5 mM *p*-nitro-phenyl-α-d-glucopyranoside solution in 0.1 M phosphate buffer (pH 7.0) was added, and the mixture was incubated at 25 °C for 5 min. After, the absorbance was recorded at 405 nm using a microplate reader. The α-glucosidase inhibitory activity was expressed as percentage inhibition and calculated as follows:%inhibition = *A*_0_ − *A*_1_/*A*_1_ × 100where: *A*_0_ was the absorbance of the control, and *A*_1_ was the absorbance in the presence of the benzimidazolone derivatives evaluated.

### 2.5 *In vitro* glucokinase activation

The GK activity of synthesized compounds was evaluated using the commercial kit “Hexokinase colorimetric assay kit” which contains 2-(4-(2-hydroxyethyl)piperazin-1-yl)ethanesulfonic acid (25 mM, pH 7.4), glucose (10 mM), KCl (25 mM) mgCl_2_ (1 mM) dithiothereitol (1 mM), adenosine triphosphate (1 mM), NAD (1 mM), glucose-6-phosphate dehydrogenase (G-6-PDH) (2.5 U mL^−1^), GK (0.5 μg), and benzimidazolones derivatives evaluated (100 and 200 μg mL^−1^); all the assays were performed in a final volume 300 μL. The absorbance of nanomoles per minute per milliliters (nmol min^−1^ mL^−1^) was measured at 450 nm after a 5 min incubation period for 60 min, the GK activation fold by benzimidazolones derivatives, control positive and normal GK fold activation (NADH standard curve) was calculated as follows:GK_act_ = *B* × sample dilution factor/(reaction time) × *V*where: *B* was the amount (nmol) of NADH generated during the reaction time, reaction time was the difference between final time and initial time; *V* was the sample volume (mL) added to well.

The results are expressed as nanomoles per minute per milliliter (nmol min^−1^ mL^−1^) and activation fold compared to the control.^16,17^

## 3 Results and discussion

### 3.1 Molecular docking analysis of benzimidazolones derivatives against pharmacologic targets

All 23 design benzimidazolones derivatives were analyzed against α-glu and GK, using Autodock Vina, in order to find the binding interaction with the active site of the targets. The affinity energy values and interactions of the best benzimidazolones derivatives in active and the allosteric site of evaluated targets are shown in Table 2. In the first case, the molecular docking assay shows affinity energy values from −8.7 to −8.6 Kcal mol^−1^. The compounds 2m and 2s, display the best *in silico* results based on their affinity energy values and the number and type of intermolecular interactions. The residues from the active site involved in bonding interaction with benzimidazolones derivatives are Asp518, Glu521, Asp616 (catalytic amino acids), Arg600, Asp282, and others; these compounds formed a hydrogen and electrostatic bond with a least 2 of the 3 catalytic amino acids reported for these targets. The compound 2m is bound into the active site of α-glucosidase by a polar hydrogen bond between N–H⋯O of Asp518 (2.54 Å, 122°), C=O⋯H–N of Arg600 (3.07 Å, 33.15°; and 3.16 Å, 35.2°) and an electrostatic bound with Asp616 (3.54 Å) *via* ring A. A polar hydrogen bond between Ar-F links the compound 2s⋯O of Arg600 (3.66 Å, 60.5°), in addition, it is bonded with Asp518 (3.20 Å and 2.94 Å) and Asp404 (3.36 Å) by a halogen interaction F⋯O of these amino acids respectively. Otherwise, the results of affinity energy values on the allosteric site of GK were from −10.9 to −9.1 Kcal mol^−1^, where the derivatives 2k, 2r, and 2s showed the best values (−9.5, −9.2, and −10.9 Kcal mol^−1^ respectively). The residues from the allosteric site involved in bonding interaction with benzimidazolones derivatives are Arg63, Pro66, His218, Ala456, Tyr214, Tyr215, Val452, Val455, and Met210. 2k derivative interacted by an electrostatic-sulphide bond with Met210 *via* ring C (5.39 Å) and hydrophobic bond with Tyr214 (3.71 and 4.91 Å) *via* ring C and A respectively BnMe⋯CH_2_ of Met210 (4.94 Å) and CH_2_ of Met235 (3.73 Å), CH of Val455 *via* ring A (4.28 Å) and BnMe⋯Ar of Tyr214 (4.23 Å). The benzylbenzimidazolone 2r display an interaction by an electrostatic-sulphide bond with Met210 *via* ring B (5.12 Å) and a hydrophobic bond with Tyr214 (4.12 and 3.66 Å) *via* rings A and B, respectively, terBu-C⋯pyrrole of Trp99 (4.85 Å) and Ar of Tyr215 (4.94 Å), terBu-Me^…^pyrrole of Trp99 (3.93 Å) and Val455 (4.39 Å) *via* ring C. The 2s binding by a polar hydrogen bond between N–H⋯O–H of Ser64 (2.67 Å, 119.35°), halogen bond Between F⋯O=C of Leu451 (3.14 Å), electrostatic-sulphide bond with Met210 (4.98 Å) *via* ring B and hydrophobic bond between rings A and B with Tyr214 (4.07 and 3.67 respectively), ring B⋯CH_3_ of Met235 (3.80 Å), ring C⋯CH_3_ of Val455 (3.45 Å) and 3F⋯CH of Leu451 (4.00 Å).

**Table 2:** Molecular docking analysis of 2k, 2m, 2r and 2s against α-glu and GK

Evaluated compounds	Affinity energy (Kcal mol^−1^) α-Glu	Affinity energy (Kcal mol^−1^) GK	Interactions
2k	−8.6	−9.1	Hydrogen bond: Trp59, Asp300 and Asp197
			Electrostatic bond: Arg195, Asp197, Glu233 and Met210
			Hydrophobic bond: Tyr214, Met210, Met235 and Val455
2m	−8.6	−9.1	Hydrogen bond: Asp518, Arg600 and Tyr377
			Electrostatic bond: Asp616, Tyr377, Asn437 and Phe433
2r	—	−9.2	Hydrogen bond: Trp59 and Asp300
			Electrostatic bond: Glu233, Met210
			Hydrophobic bond: Tyr215, Trp99 and Val455
2s	−8.7	−10.9	Hydrogen bond: Arg600, Asp518 and Asp404
			Electrostatic bond: Met210
			Hydrophobic bond: Tyr214, Met235 and Val455

**Fig. 2:**
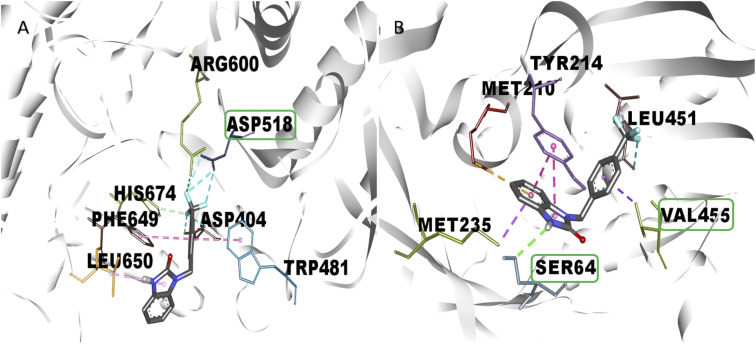
Interaction of 2r in the active (α-glu) or allosteric site (gk) of therapeutic targets. (a) Bond formation in active site of α-glu, where there are halogen bonds with one catalytic residue, (b) bond formation in the allosteric site of gk, where present interaction with two biologically relevant residues. In both cases, the green lines represent the hydrogen bond; the orange lines represent the electrostatic bonds and purple and pink lines represent the hydrophobic bonds.

One of the main aspects in the discovery and generation of new drugs, is the design of specific and effective structures against a pharmacologic target, using atoms and functional groups that have been reported with biological relevance against pharmacologic targets related to the control of hyperglycemia states like α-glu and GK.^13,27^ Therefore, *in silico* evaluation play a critical role in identifying novel molecules that can control blood glucose levels, using molecular docking to show the interactions with biological relevance amino acids in the union site, which helps to predict the possible biological activity.^28^ The molecular docking analysis suggests good affinity energy (values < −5 Kcal mol^−1^); these values could be explained in the amino acid residues interaction, where the evaluated compounds present hydrogen, electrostatic and hydrophobic bond with catalytic or biological important residues of the active or allosteric site. Some reports in the literature suggest that binding with this catalytic triad like Asp518, Glu521, Asp616 (α-glu) and Val452, Tyr377, Asn437, and Phe43 (GK) are crucial in the inhibition and/or activation of α-glu and GK respectively.^16,29^ Compound 2s (the benzimidazolone with the best docking profile), presented interaction whit all the targets evaluated, showing interaction with at least one catalytic residue. In α-glu, it presents a hydrogen bond with Asp518, and in GK shows hydrogen and hydrophobic interaction with Ser64 and Val455; these type of bonds explain the good affinity energy values (Fig. 2). Currently, there are no docking studies of benzimidazolones derivatives against GK, and only a few against α-glu; however, Mentense *et al.* in 2020, Aispuro-Pérez *et al.* in 2019, Kadhase *et al.* in 2019 and Singh *et al.* in 2019, performed studies of heterocyclic compounds with structural similarity than benzimidazolones; their results showed affinity energy values lower than these derivatives (−11.1 to −8.0 Kcal mol^–1^), which suggest that the evaluated compounds have better potential than the compound reported by these authors.^4,8,25,27^

Based on the *in silico* evaluation (affinity energy values and interaction with catalytic amino acids) and the good potential showed of benzimidazolones derivatives compared with heterocyclic compounds reported by different authors; four benzimidazolone derivatives were selected to be synthesized (2k, 2m, 2r and 2s), which present the benzyl group as a substituent in the heterocyclic nitrogen.

### 3.2 Synthesis of benzylbenzimidazolones derivates

Continuing with our research interest, we report the synthesis of the compounds 2k, 2m, 2r, and 2s, by a conventional heat and catalysis-free reaction of the benzimidazolone core (1,3-dihydro-2*H*-benzo[*d*]imidazole-2-one) obtained by microwave irradiation. This protocol is an alkylation with benzyl bromides to obtain the monosubstituted benzimidazolone. Therefore, the initial tests were devoted to finding the best reaction conditions. For this, compound 2k was studied as a reaction model. The first assays were carried out at room temperature for 72 hours with DMF as solvent and sodium hydride as a base. After the reaction time, the desired compound 2k was isolated with a yield of 9%. In addition, the disubstituted compound was isolated with a yield of 15%; these compounds were isolated by a chromatographic column. Then, a variation in temperature (60–70 °C) and a decrease in time (72 to 24 h) showed an increase in yield of up to 27% (Table 3). In these experiments, set the best conditions were set at 70 °C and 24 h. To delimit the obtaining of the monosubstituted benzimidazolone; the synthesis process was monitored by thin layer chromatography (TLC), taking samples of the reaction every hour, then it was observed the formation of both compounds in the first hour of the reaction, showing a high rate of formation of the disubstituted compound. After that, to decrease the reaction speed, it was used potassium carbonate instead of sodium hydride, obtaining a higher yield of 38% in monosubstituted and 42% in disubstituted benzimidazolone (Table 4). The benzimidazolones derivatives 2k, 2m, 2r, and 2s were prepared using the conditions previously described in 22–38% yields (Table 4), and their structural confirmation was performed by gas chromatography/mass spectrometry (GC-MS) and nuclear magnetic resonance (NMR) analysis (ESI†). The GC-MS of 2k showed the presence of the quasi-molecular ion *m*/*z* = 239.1 [M + H]^+^ at 25.5 min. Otherwise, the ^1^H-NMR spectrum showed characteristic signals for the molecular structure; for example, at 10.1 ppm it is observed a singlet that corresponds to the H–N in the imidazole ring, a singlet at 4.98 ppm is associated with 2H in the benzylic methylene and a singlet at 2.23 ppm associated to 3H in the substituent in the benzylic ring. The ^13^C-NMR spectrum presents signals at 156.0, 44.5, and 21.2 ppm corresponding to the carbonyl, benzylic methylene, and methyl carbon, respectively. These signals are important because they corroborate the alkylation of benzimidazolone core. The results obtained in this synthesis are similar to those reported by Abbas *et al.* in 2015, where the confirmation of synthesized benzimidazolones derivatives with ^1^H-NMR showed a signal of H–N proton in the range 11.50–9.75 ppm; at the same time, the ^13^C-NMR analysis presents a signal of the carbonyl group in the range of 152.0–148.0 ppm.^5^ Therefore, these results could be important to the data generating of these compounds.

**Table 3:** Standardization of synthesis route of benzylbenzimidazolone derivatives

Benzyl benzimidazolone	Experimental mass (mg)	Yield (%)	Reaction conditions
2k	30	19	Room temperature/72 h
	42.2	22	60 °C/24 h
	60	27	70 °C/24 h

**Table 4:** Yields and mass obtained in synthesis of benzylbenzimidazolone derivatives 2k, 2m, 2r and 2s

Benzimidazolone	Experimental mass (mg)	Yield (%)
2k	100	38
2m	44.6	23
2r	65.1	26
2s	37.8	22

One of the important results obtained in the synthesis of benzylbenzimidazoles was the formation of disubstituted compounds with better yields than the monosubstituted molecules; this could be explained by the highest reactivity of monosubstituted benzylbenzimidazolone, which causes the reaction of the alkylating agent with this intermediate instead of the benzimidazolone core. Currently, some authors report synthesis pathways for benzimidazolone derivatives with structural similarity protection of the amino group for a later sulfonation at 20–140 °C.^5^ Here, the experimental conditions were suitable to generate benzimidazolones derivatives in fewer steps at a lower temperature; showing a less complicated synthesis of these compounds.

### 3.3 α-glucosidase inhibitory activity

The enzymatic inhibition against α-glu of the 2k, 2m, 2r, and 2s derivatives are shown in Table 5. These compounds were evaluated at 0.1–100 μg mL^−1^ using acarbose as a reference standard. Analyzing the inhibition results, these compounds present percentage values from 7 ± 0.9 to 36 ± 0.30% with a concentration-dependent behavior. The compound 2k displays an inhibition range of 14 ± 1.23 to 29 ± 0.45; 2m present 12 ± 2.21 to 36 ± 0.30, 2r showed 7 ± 2.21–13 ± 1.34 and 11 ± 0.74 to 35 ± 2.95 for 2s compound, where the compounds 2m and 2s present the best inhibition values; nevertheless, compared to the reference drug (6 ± 1.30 to 57 ± 0.70) our benzylbenzimidazolones derivatives present moderate inhibition against α-glu.

**Table 5:** Inhibition activity of benzylbenzimidazolones derivatives 2k, 2m, 2r, and 2s in an *in vitro* evaluation on α-Glu

Inhibition percentage of benzylbenzimidazolones derivatives					
Evaluated compounds	% Inhibition 0.1 μg mL^−1^	% Inhibition 1 μg mL^−1^	% Inhibition 10 μg mL^−1^	% Inhibition 50 μg mL^−1^	% Inhibition 100 μg mL^−1^
2k	14 ± 1.23	20 ± 0.89	21 ± 0.94	29 ± 0.45	—
2m	12 ± 2.21	27 ± 1.98	35 ± 0.49	36 ± 0.30	—
2q	—	7 ± 0.91	13 ± 1.34	—	—
2r	—	—	—	11 ± 0.74	35 ± 2.95
Acarbose (drug reference)	—	6 ± 1.30	30 ± 5.20	43 ± 6.7	57 ± 0.70

**Fig. 3:**
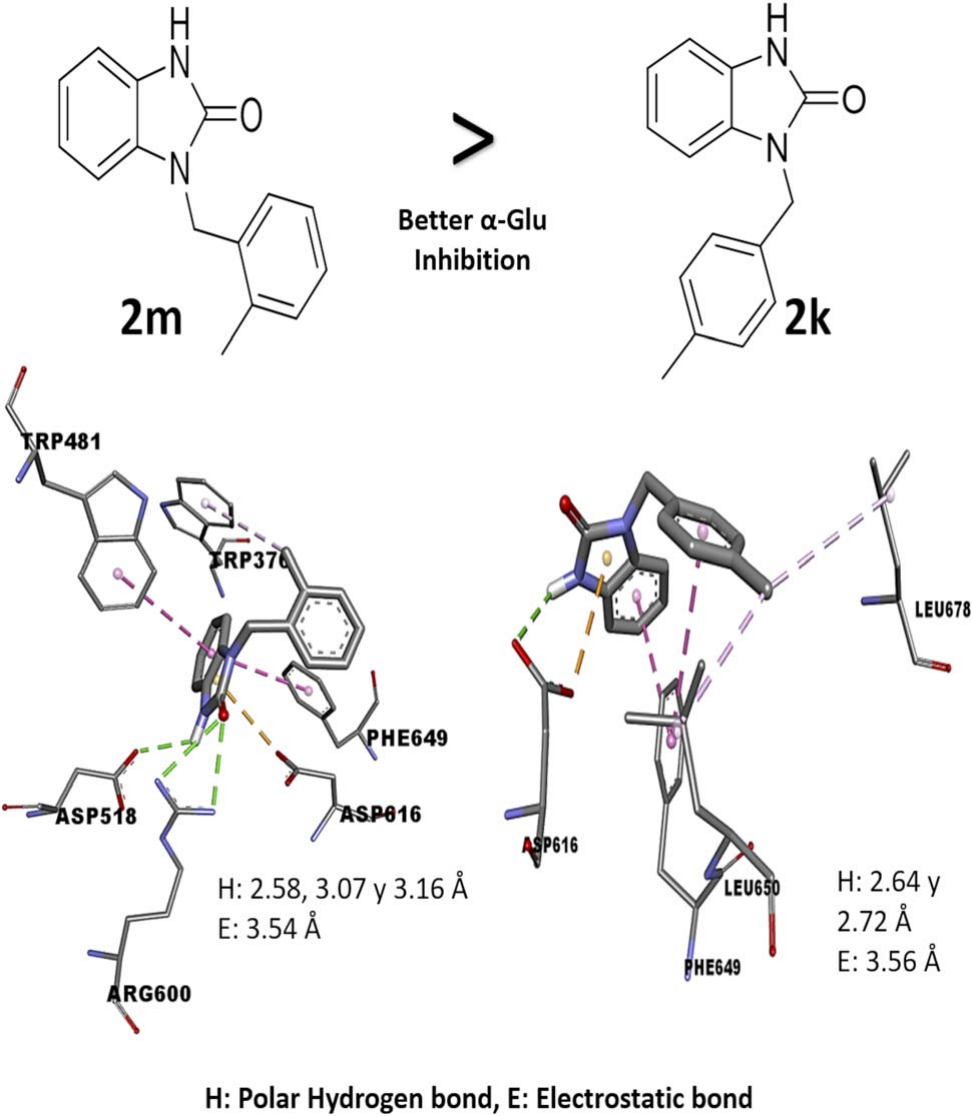
Structure–activity relationship analysis of 2m and 2k compound against α-glu with *in silico* interaction. The interaction in 2m compound shows a hydrogen and electrostatic bond with shorter distance (2.58, 3.16 and 3.54 Å) than bonds in the compound 2k (2.64, 3.72 and 3.56 Å), also, these bonds are with two catalytic amino acids.

Comparing the results of 2k and 2m, which have the same alkylating group with different orientations (*p*-methylbenzyl and *o*-methylbenzyl, respectively), it is observed that 2m has better *in vitro* activity than 2k; the analysis of their interactions by molecular docking helps to explain their difference in biological activity. The 2m compound presents three polar hydrogen bonds (2.58, 3.07, and 3.19 Å of distance) and one electrostatic bond (3.54 Å) with Asp518 and Asp616 (catalytic amino acids). On the other hand, 2k presents two polar hydrogen bonds (2.64 and 2.72 Å of distance) and one electrostatic bond (3.56 Å) with a catalytic amino acid (Asp616); these differences in distances and interaction with catalytic amino acid could explain the *in vitro* inhibitory activity (Fig. 3). Nowadays, there are few reports of *in vitro* evaluation of benzimidazolones against α-glu; for example, Menteşe *et al.*, in 2021, evaluated benzimidazolones attached to tri-heterocycles, showing inhibition percentage of 60–92% at 100 μg mL^−1^. Comparing the results in our work with the reported by this group; suggests that benzimidazolones derivatives with low or high molecular weight can present good affinity and inhibition against intestinal enzymes like α-glu.^30^

### 3.4 *In vitro* glucokinase activation

In the case of the GK activation by benzylbenzimidazolones derivatives, an *in vitro* assay was performed using Hexokinase colorimetric assay kit, showing the determination of nanomoles per minute per milliliters (nmol min^−1^ mL^−1^) of NADH generated by GK. The evaluation of 2k, 2m, 2r, and 2s were performed at 0.1–200 μg mL^−1^; giving 23 to 86 nmol NADH at 100 and 200 μg mL^−1^. On the other hand, the positive control presented 66 and 75 nmol, respectively, while the standard of NADH showed 23 nmol (Table 6). Comparing the nmol of NADH generated by benzylbenzimidazolones derivates with the normal enzymatic reaction, compound 2k increases the enzymatic activity 1.31 and 2.38-fold, 2m increases 2.13-fold (at 200 μg mL^−1^) and 2s increases 2.86 and 3.74-fold at 100 and 200 μg mL^−1^ respectively. These results showed that the compound 2s presented the best activation potential of GK, even better than the positive control including in the Hexokinase colorimetric assay kit (an increase of 3.26-fold).

**Table 6:** *In vitro* evaluation of activation potential of benzylbenzimidazolone derivatives 2k, 2m, 2r and 2s against GK

Activation fold of GK of benzylbenzimidazolone derivatives				
Evaluated compounds	nmol min^−1^ mL^−1^ of NADH a 100 μg mL^−1^	nmol min^−1^ mL^−1^ of NADH a 200 μg mL^−1^	Activity increase	
			100 μg mL^−1^	200 μg mL^−1^
2k	30	65	1.31	2.83
2m	—	49	—	2.13
2q	—	—	—	—
2r	45	86	1.96	3.74
Positive control	66	75	2.86	3.26
NADH standard	23			

**Fig. 4:**
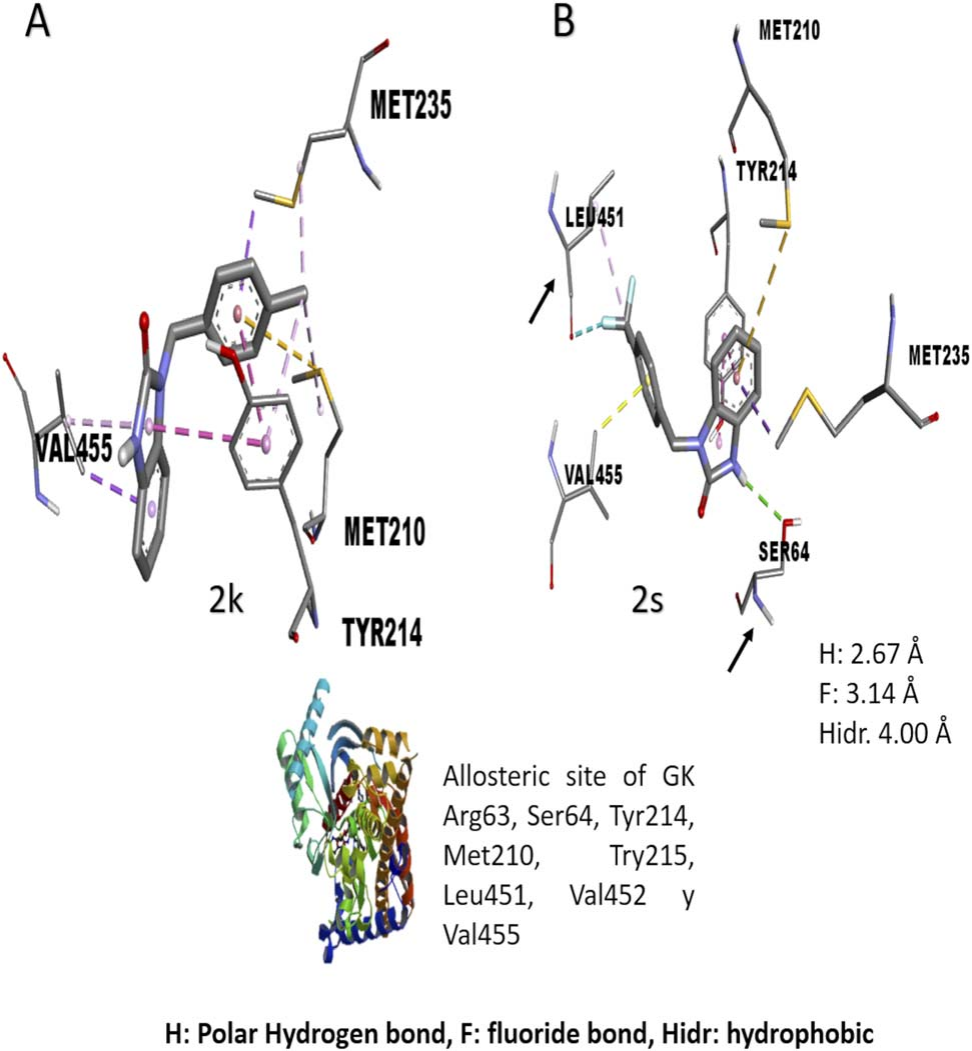
Structure–activity relationship analysis of 2k and 2s compounds against gk with *in silico* interaction. Both of benzylbenzimidazolones derivatives shows interaction in the allosteric site of gk. (a) Interaction of 2k compound showed hydrophobic interaction 4 amino acid of the allosteric site. (b) Interaction of 2s compound showed hydrogen and fluorine interactions with extra amino acids like ser64 and leu451 respectively.

The *in vitro* result against this enzyme showed that 2k and 2s have a high enzymatic activation; a structure–activity relationship assay using the interactions in molecular docking of these compounds (Fig. 4) shows that 2k derivative presents hydrophobic interactions with biologically relevant amino acids in the allosteric site reported (Tyr214, Met210, and Val455). However, the 2s derivative presents polar hydrogen, fluoride and hydrophobic bonds (2.67, 3.14 y 4.0 Å of distance) with other amino acids like Leu451 and Ser64 respectively, suggesting that these interactions may be responsible for their enzymatic activation.^31,32^ Nowadays there are no studies of *in vitro* evaluation of benzimidazolones as activators of GK; however, authors like Charaya *et al.*, in 2018, evaluated heterocyclic compounds such as thiazolino benzamides as activators of GK, showing an increase enzymatic activity of 1.48 to 1.83-fold, comparing the benzylbenzimidazolones derivatives they present a better enzymatic activation potential of GK than thiazolin benzamides.^16^

## 4 Conclusions

**Fig. 5:**
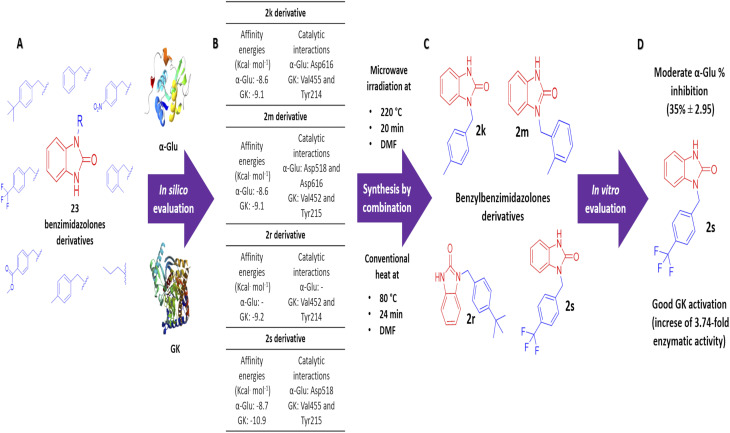
Flow chart of the strategy used in the generation of benzimidazolones derivatives with blood glucose control potential. (a) Design of benzimidazolones derivatives using electron withdrawing groups as substituents. (b) Selection of 4 benzimidazolones derivatives with benzyl group as substituent based on the affinity energy values and catalytic interactions. (c) Synthesis of 4 benzylbenzimidazolones derivatives under a microwave irradiation/conventional heat combination. (d) Selection of the 2s derivative as the compound with the best *in vitro* potential as blood glucose control drug.

The design and *in silico* evaluation of the 23 benzimidazolones derivatives shows that 2k, 2m, 2r, and 2s (benzyl group as a substituent) present the best *in silico* profile; showing better affinity energy than reference drugs evaluated and interaction with biological relevance amino acids of α-glu and GK. A novel method of synthesis of benzylbenzimidazolone based on microwave irradiation/conventional heat combination is reported. These compounds showed moderated inhibition against the intestinal enzyme and a good activation of GK, where the 2s compound showed good potential as a blood glucose control drug since presenting an increase of GK (2.86 and 3.74-fold at 100 and 200 μg mL^−1^) better than the positive control (an increase of 3.26-fold) (Fig. 5). These results suggest that these compounds could be a good candidate to keep studying with biological evaluations; generating a complete pharmacologic and toxicological profile.

## Author contributions

C. L. Santos-Ballardo: conceptualization; data curation; formal analysis; investigation; methodology; software; visualization; writing – original draft. J. Montes-Ávila: data curation; formal analysis; funding acquisition; investigation; resources; supervision; validation; writing – original draft; writing – review & editing. J. G. Rendon-Maldonado: investigation; writing – review & editing. R. Ramos-Payan: resources; writing – review & editing. S. Montaño: supervision; writing – review & editing. J. I. Sarmiento-Sánchez: conceptualization; data curation; formal analysis; funding acquisition; investigation; methodology; resources; software; supervision. S. J. Acosta-Cota: supervision; writing – original draft; writing – review & editing. A. Ochoa-Terán: formal analysis; methodology; resources; writing – review & editing. P. J. Bastidas-Bastidas: formal analysis; methodology; resources, validation. U. Osuna-Martínez: conceptualization; formal analysis; funding acquisition; investigation; methodology; project administration; resources; supervision; validation; writing – original draft; writing – review & editing.

## Conflicts of interest

The authors have no potential conflicts of interest to report.

## Supplementary Material

RA-013-D3RA02916F-s001

## References

[cit1] Ronald P., Jhon C., Louise E., Andrea H., Philip J., Alan P., Grant W., Kazuya Y. (2008). Bioorg. Med. Chem..

[cit2] Anna-Maria M., Patrizia L., Laura D. L., Nunzio I., Stefania F., Giovanni M., Erik D. C., Chirstophe P., Alba C. (2010). Bioorg. Med. Chem..

[cit3] Deepak N., Bhalchandra B. (2015). Green Chem..

[cit4] Emre M., Nimet B., Mustafa E. (2020). Bioorg. Chem..

[cit5] Muhammad A., Shahid H., Muhammad F., Jörg K., Nasir M. (2015). Bioconjugate Chem..

[cit6] Sana I., Ameni G., Nesrine A., Hasan M., Melek H., Radhouane B. H. T., Moncef M. (2021). Monatshefte fur Chemie.

[cit7] Osmeri S. A., Octavio D. G., Leobardo G. O., Leticia L. M. (2013). CienciaUAT.

[cit8] Saurab K., Nikhil A., Krushna D., Ashish D., Vikas P., Deepak L., Vinod U., Nitin C., Vivekanand C. (2020). J. Mol. Struct..

[cit9] Yan-Fei Z., Zhi-Rong L., Li Y., Sangho O., Jun-Mo Y., Jinhuk L., Zhuo Y. (2012). Process Biochem..

[cit10] Rodley M., Sheri H., AnQiang Z., Peter V., Robert C., Michael B., Beverly M., Mariko K., William C. (2012). Gene.

[cit11] Kritika S., Praveen T., Vinay S., Ashok P., Srivastava O. N., Singh S. K., Arvind K. (2019). Int. J. Pept. Res. Ther..

[cit12] Dasha M., Ivelina D., Aneta P., Ivayla D., Radkka V., Anna L., Albert K. (2021). Molecules.

[cit13] Manoj D., Preeti G. (2019). Eur. J. Med. Chem..

[cit14] Nikhil J., Akshata P., Neha D., Vikas T. (2017). Med. Chem. Res..

[cit15] Fazal R., Fazal M., Hayat U., Abdul W., Fahad K., Muhammad J., Muhammad T., Wajid R., Ashfaq R., Khalid K. (2015). Bioorg. Chem..

[cit16] Neha C., Deepti P., Ajmer G., Viney L. (2018). Comput. Biol. Chem..

[cit17] Ajmer G., Rajeev K., Deo P., Jagdeep D., Viney L. (2019). Chem. Biol. Drug Des..

[cit18] Yixin R., Li L., Li W., Yan H., Shuang C. (2022). J. Enzyme Inhib. Med. Chem..

[cit19] Konstantinos T., Krishnaraj N., Chrysa P., Anthony B., Abd T. (2020). Drugs.

[cit20] Amin N. B., Aggarwal N., Pall D., Paragh G., Denney W. S., Le V., Riggs M., Calle A. (2015). Diabetes, Obes. Metab..

[cit21] kiyosue A., Hayashi N., Komori H., Leonsson-Zachirisson M., Johnsson E. (2013). Diabetes, Obes. Metab..

[cit22] Saurab K., Nikhil A., Manasi D., Deepak L., Ravindra P., Vinod U., Nitin C., Vivekanand C. (2019). Future J. Pharm. Sci..

[cit23] Veronica S., Stefano M. (2018). Front. Pharmacol..

[cit24] Ehsan M., Asif J., Amina S., Shahzad M., Muhammad Z., Bilal K., Sajjad S., Muhammad T., Kanwal, Khalid K. (2018). Bioorg. Med. Chem..

[cit25] Analy A., Juan L., Fernando G., Julio M., Lorenzo P., Adrián O., Pedro B., Sarita M., Loranda C., Ulises O., Juan S. (2020). Bioorg. Chem..

[cit26] Lumadhar S., Fernando G., Lorenzo P., Adrián O., Pedro B., Loranda C., Guadalupe R., Ulises O., Juan S. (2020). J. Chem. Sci..

[cit27] Mishra S., Dahima R. (2019). J. Drug Delivery Ther..

[cit28] Rubina B., Dharam P., Garima K., Asif H., Ravi K., Azhar I. (2018). Bioorg. Chem..

[cit29] Zhiyang L., Shutao M. (2017). ChemMedChem.

[cit30] Emre M., Okan G., Nedime C., Nimet B. (2021). J. Heterocycl. Chem..

[cit31] Ajmer G., Sandeep A., Neelam S., Sukhbir S. (2020). Plant Archives.

[cit32] Ammar H., Omar A. R., Monther M., Abdul-Jabbar A. (2022). International Journal of Drug Delivery Technology.

