# Supplementary material for: Design, synthesis, in silico, and in vitro evaluation of benzylbenzimidazolone derivatives as potential drugs on α-glucosidase and glucokinase as pharmacological targets
Source: RSC Adv. 2023 Jul 12;13(31):21153–62. doi: 10.1039/d3ra02916f (PMC10337652; doi:10.1039/d3ra02916f)
Supplement: RA-013-D3RA02916F-s001 [file RA-013-D3RA02916F-s001.pdf]

Electronic Supplementary Material (ESI) for RSC Advances.
This journal is © The Royal Society of Chemistry 2023

# Design, synthesis, in silico, and in vitro evaluation of benzylbenzimidazolones derivatives as potential drugs on α-glucosidase and glucokinase as therapeutic targets

**Santos-Ballardo Cress Lumadhar[a], Montes-Ávila Julio[a*], Rendon-Maldonado José Guadalupe[a], Ramos-Payan Rosalio[a], Montaño-Valdez Marisela Sarita[a], Sarmiento-Sánchez Juan I.[b†], Acosta-Cota Selene de Jesús[c], Ochoa-Terán Adrián[d], Bastidas-Bastidas Pedro de Jesus[e], Osuna-Martínez Ulises[a*].**

[a] Facultad de Ciencias Químico Biológicas, Universidad Autónoma de Sinaloa, México.

[b] Facultad de Ingeniería Civil, Universidad Autónoma de Sinaloa, México.

[c] Departamento de Ciencias de la Salud, Universidad Autónoma de Occidente, México.

[d] Centro de Graduados e Investigación en Química, Instituto Tecnológico de Tijuana, México.

[e] Laboratorio de Análisis de Residuos de Plaguicidas, Centro de Investigación en Alimentación y Desarrollo A.C., México

* Corresponding authors
🖂 Ulises Osuna Martínez
ulises.osuna@uas.edu.mx
🖂 Julio Montes-Avila
jmontes@uas.edu.mx

## Table of Contents

## $^{1}$H-NMR, $^{13}$C-NMR and GC-MS of benzylbenzimidazolones:

**Figure 1S.** $^{1}$H NMR spectrum of compound **2k** .......... 3
**Figure 2S.** $^{13}$C NMR spectrum of compound **2k** .......... 3
**Figure 3S.** GC-MS of compound **2k** .......... 4
**Figure 4S.** $^{1}$H NMR spectrum of compound **2m** .......... 4
**Figure 5S.** $^{13}$C NMR spectrum of compound **2m** .......... 5
**Figure 6S.** GC-MS of compound **2m** .......... 5
**Figure 7S.** $^{1}$H NMR spectrum of compound **2r** .......... 6
**Figure 8S.** $^{13}$C NMR spectrum of compound **2r** .......... 6
**Figure 9S.** GC-MS of compound **2r** .......... 7
**Figure 10S.** $^{1}$H NMR spectrum of compound **2s** .......... 7
**Figure 11S.** $^{13}$C NMR spectrum of compound **2s** .......... 8
**Figure 12S.** GC-MS of compound **2s** .......... 8

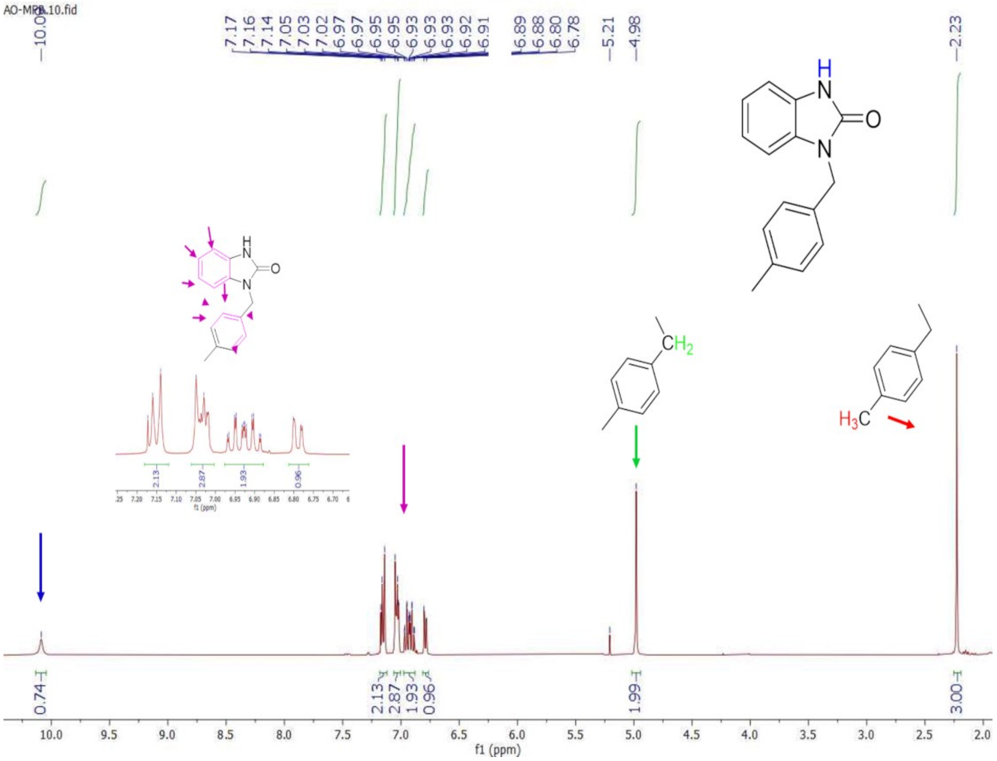


**Figure 1S.** $^{1}H$ NMR spectrum of compound **2k**

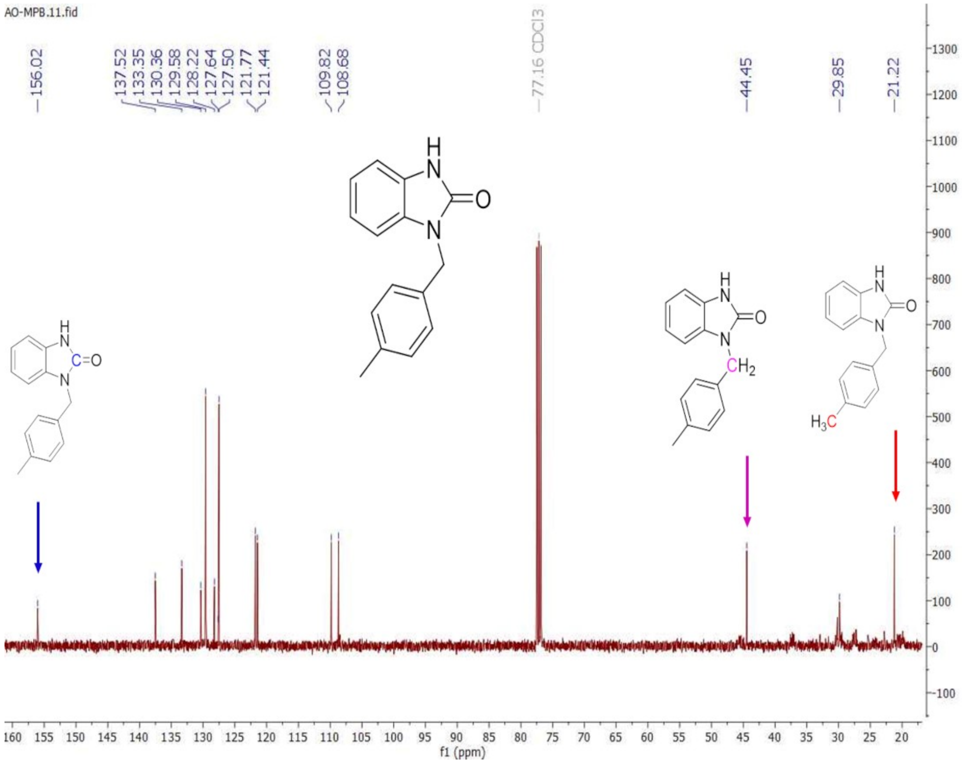


**Figure 2S.** $^{13}C$ NMR spectrum of compound **2k**

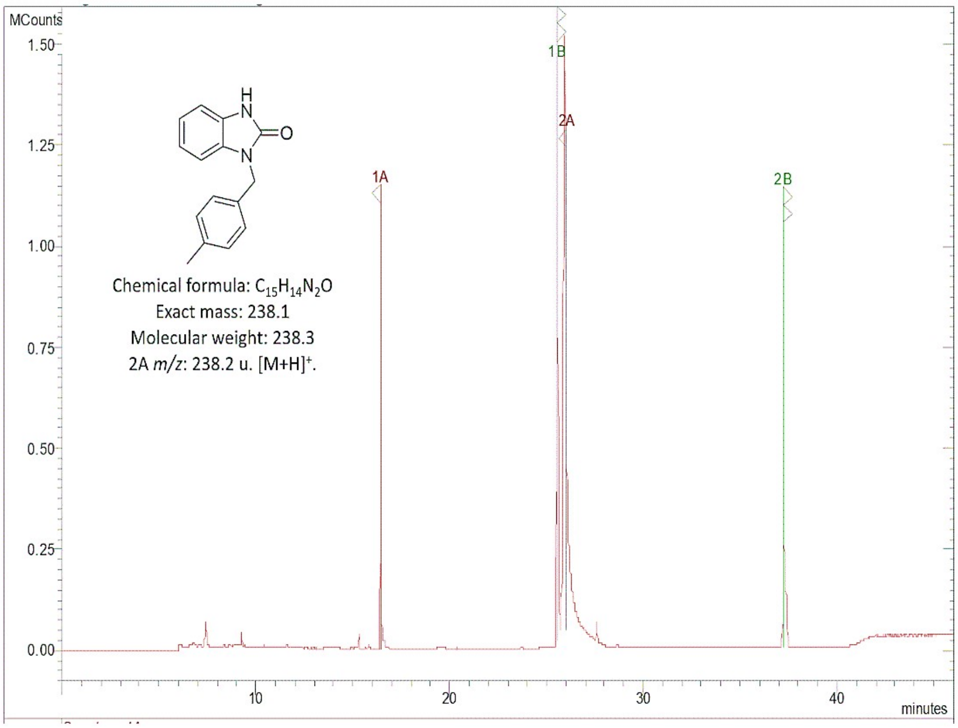


**Figure 3S.** GC-MS of compound **2k**

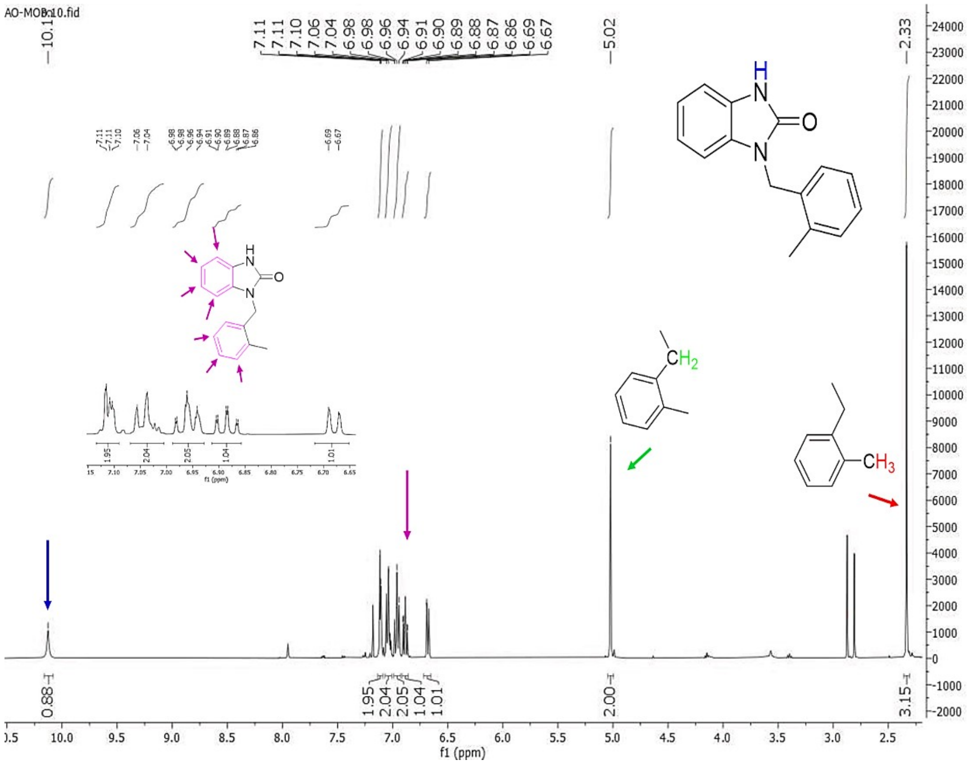


**Figure 4S.** $^{1}$H NMR spectrum of compound **2m**

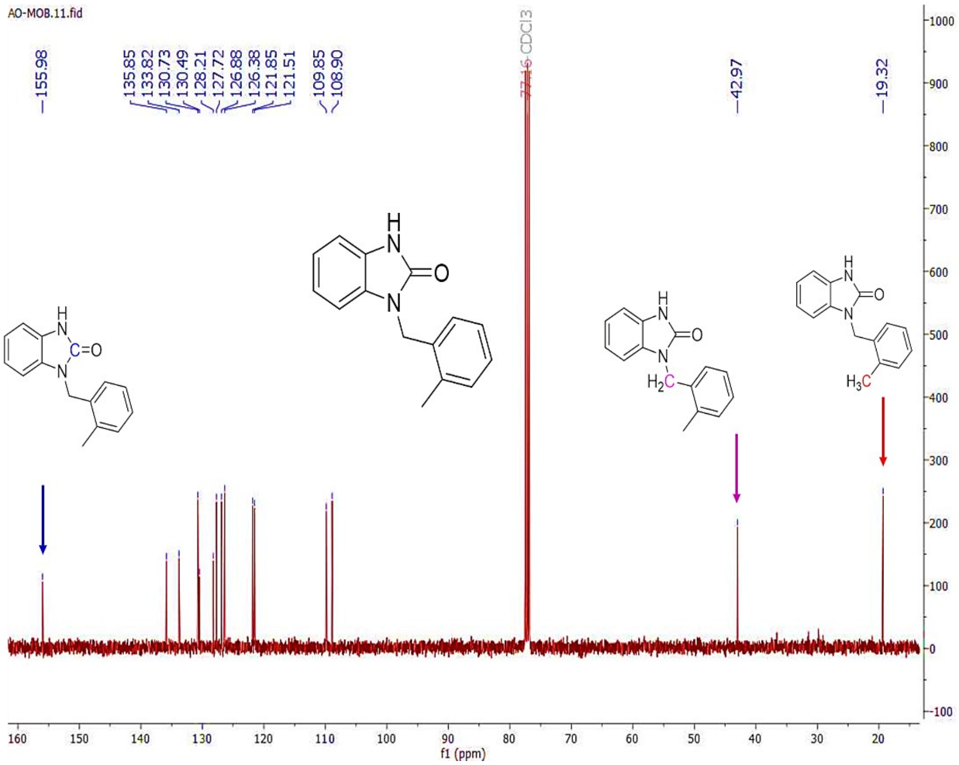


**Figure 5S.** $^{13}C$ NMR spectrum of compound **2m**

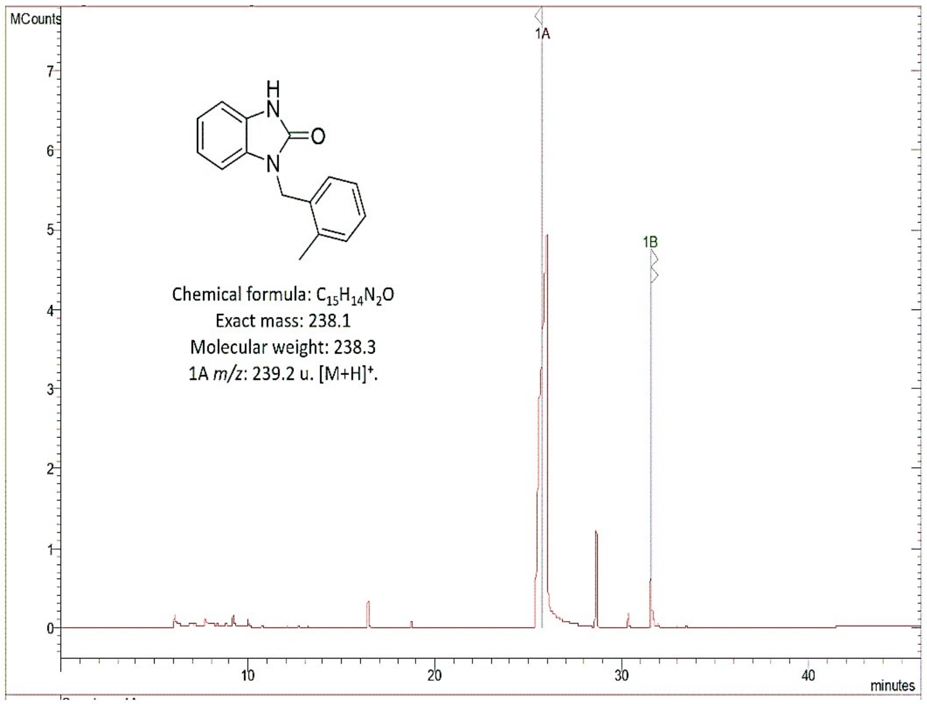


**Figure 6S.** GC-MS of compound **2m**

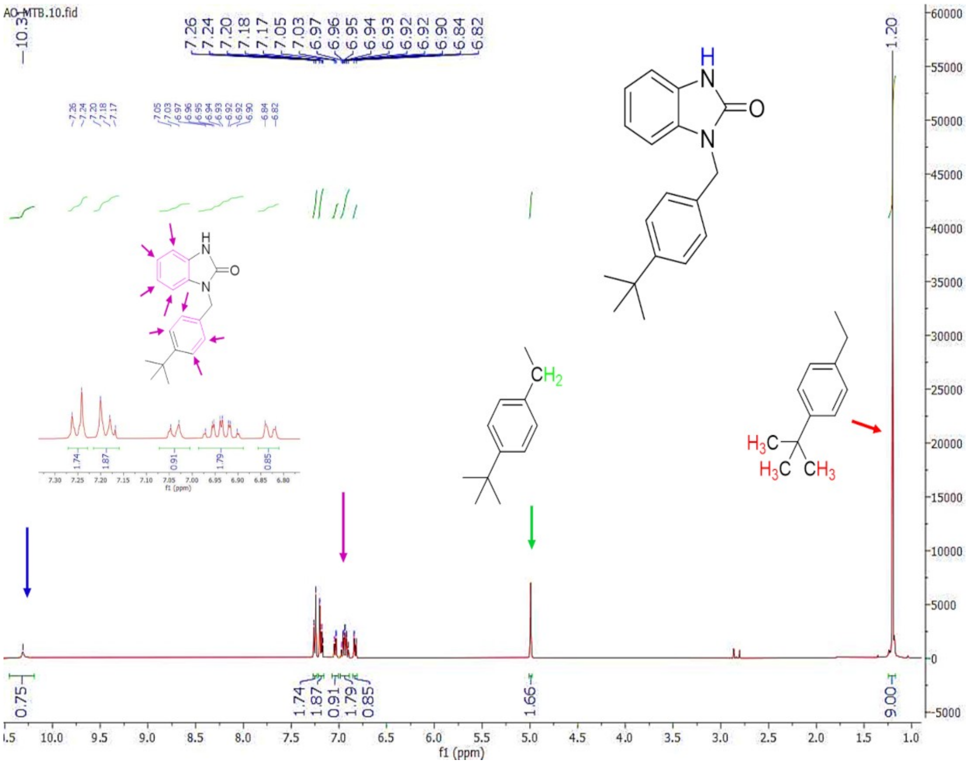


**Figure 7S.** $^{1}H$ NMR spectrum of compound **2r**

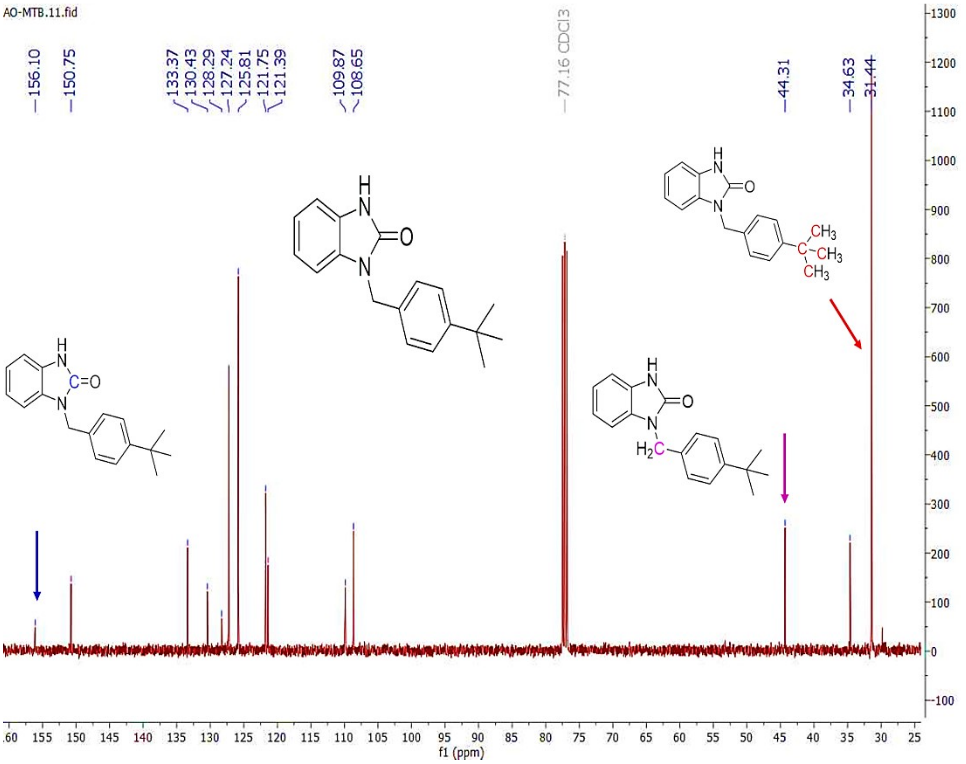


**Figure 8S.** $^{13}C$ NMR spectrum of compound **2r**

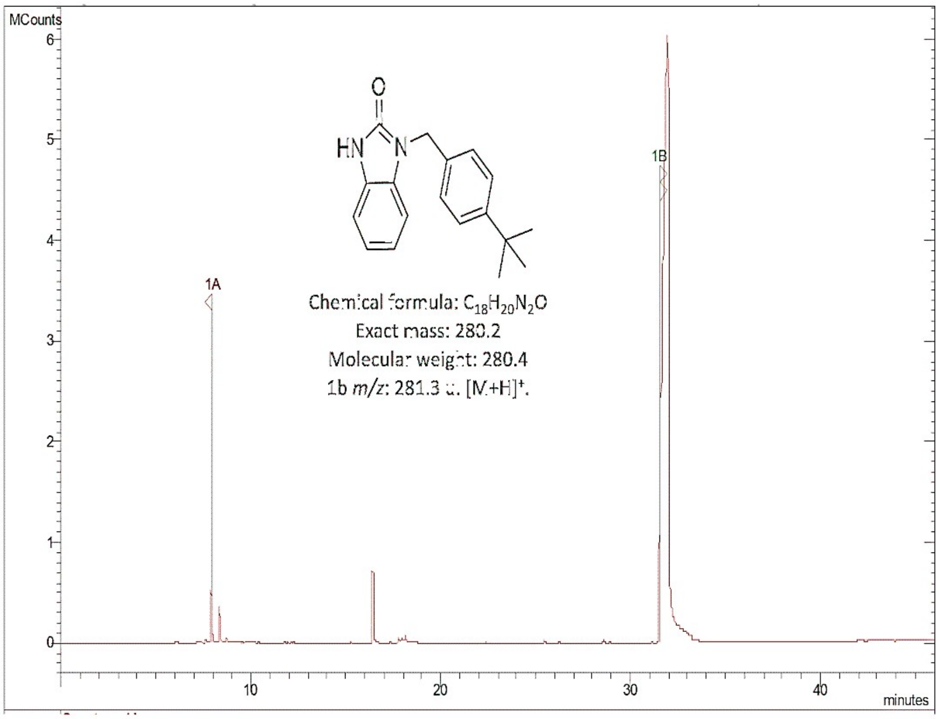


**Figure 9S.** GC-MS of compound **2r**

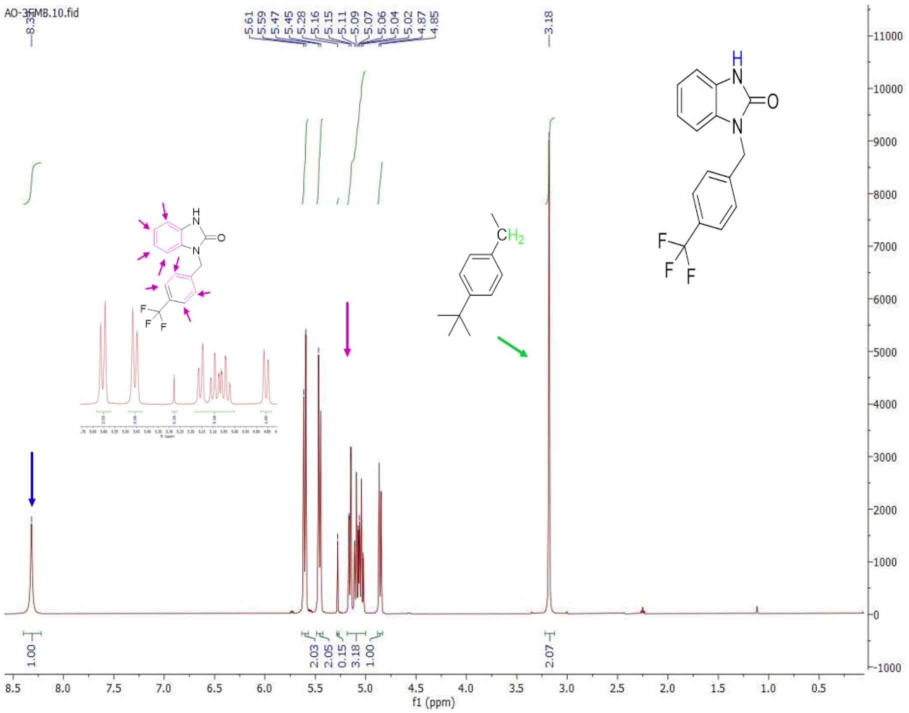


**Figure 10S.** $^{1}$H NMR spectrum of compound **2s**

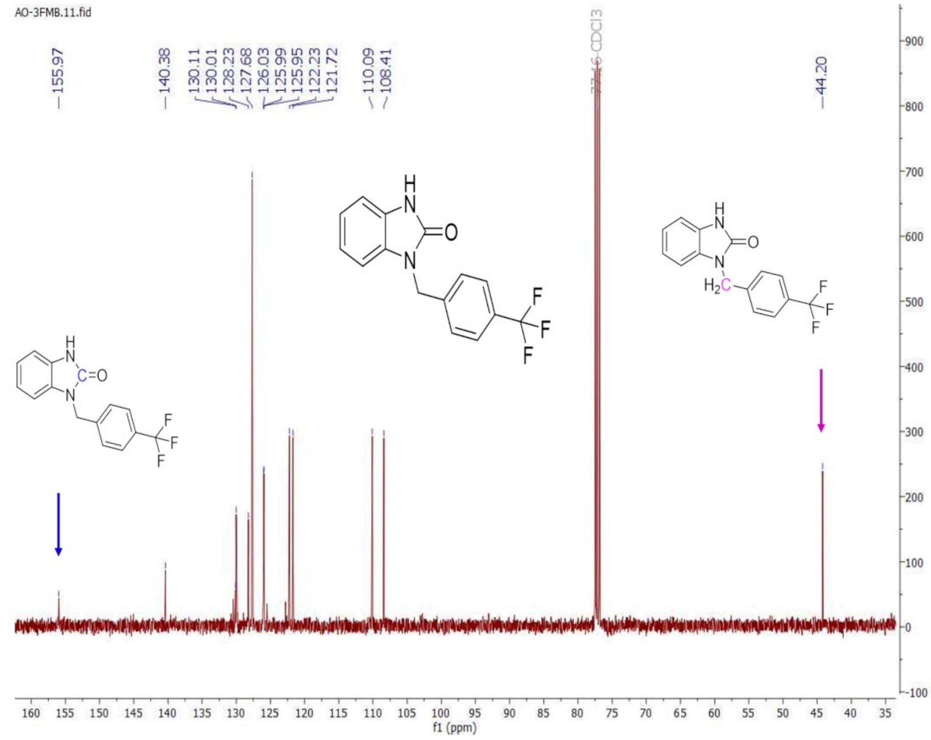


**Figure 11S.** $^{13}$C NMR spectrum of compound **2s**

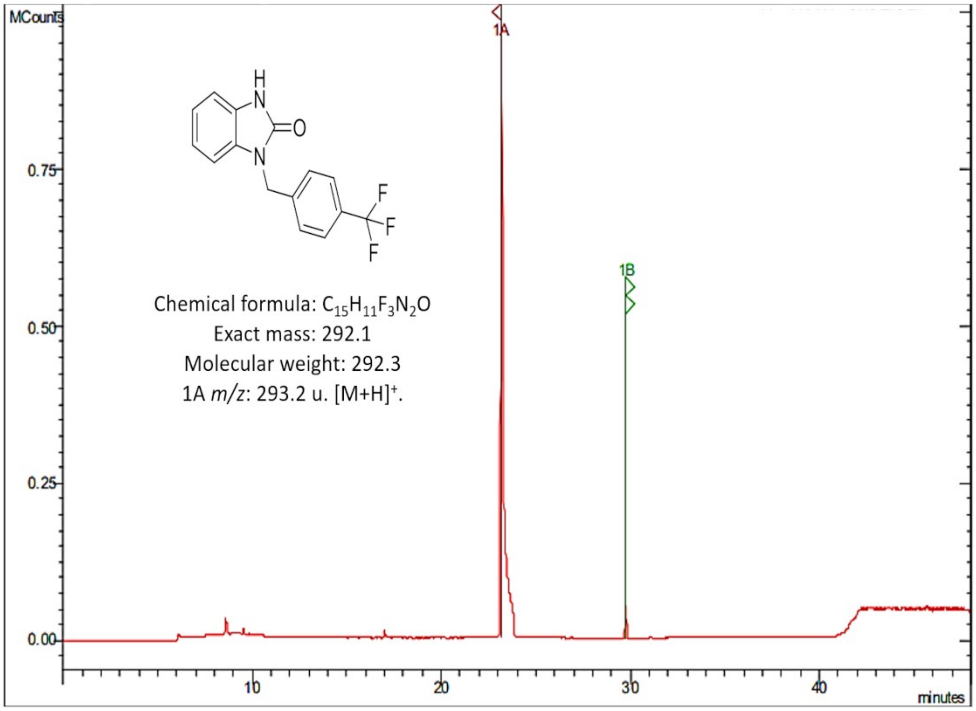


**Figure 12S.** GC-MS of compound **2s**
